# Delayed presentation of severe combined immunodeficiency due to prolonged maternal T cell engraftment

**DOI:** 10.4103/0256-4947.62834

**Published:** 2010

**Authors:** Saleh Z. Al-Muhsen

**Affiliations:** From the Department of Pediatrics, King Khaled University Hospital, King Saud University and the Department of Pediatrics, King Faisal Specialist Hospital and Research Center, Riyadh, Saudi Arabia

## Abstract

Severe combined immunodeficiency (SCID) is a primary immunodeficiency disorder with heterogenous genetic etiologies. We describe a typical case in a 9-year-old boy that was masked by a clinically functional maternal T cell engraftment leading to late presentation with *Pneumocystis jiroveci* pneumonia and cytomegalovirus infection, probably following exhaustion of maternally engrafted cells. Based on immunological findings, he had a T- B+SCID phenotype. This report suggests that in rare cases, engrafted maternal T cell might persist for long time leading to partial constitution of immune function and delayed clinical presentation of SCID.

Severe combined immunodeficiency (SCID) is a primary immunodeficiency disorder with heterogeneous genetic etiologies, characterized by a profound defect in both T and B lymphocytes.^1^,^2^ Affected individuals usually present in early infancy with severe and persistent infections.^3^ Without hematopoietic stem cell transplantation (HSCT) or gene therapy, most patients die in early childhood.^4^,^5^ Transplacentally derived maternal T lymphocytes are frequently detected in healthy newborns; however, they are rapidly eliminated by immune competent T lymphocytes.^6^,^7^ On the contrary, SCID infants do not usually reject maternally engrafted cells; therefore maternal T cells were detected in 24% to 40% of patients undergoing hematopoietic stem cell transplantation.^8^,^9^ Since these T cells are usually non-functional, they do not alter the course of the disease and patients present typically in early infancy with severe infection.^9^ We present a typical case of SCID masked by a clinically functional maternal T-cell engraftment leading to late presentation of the disease at the age of 9 years with *Pneumocystis jiroveci* pneumonia (PJP) and cytomegalovirus (CMV) infections, probably following exhaustion of maternally engrafted T lymphocytes.

## CASE

A 9-year-old Saudi Arabian boy was referred to our hospital for further investigation of slowly resolving pneumonia. He was a product of full-term, uneventful pregnancy, with good birth weight and had an unremarkable neonatal period. Apparently, he remained healthy until the age of 5 years when he started to have recurrent attacks of cough and dyspnea, which were treated with bronchodilator and prophylactic steroid in addition to frequent use of oral antibiotics, with good response. Six months prior to presentation to our hospital, he started to show gradual clinical deterioration. He presented with lower respiratory tract infection not responding to several courses of oral antibiotics. He had no history of recurrent otitis media or sinusitis, no history of skin abscesses, dermatitis or any other skin lesions, and no history of chronic diarrhea. He received all vaccinations as per routine schedule with no apparent complications. The parents were first-degree cousins, but apart from the atopy which both parents have, there was no history of immune deficiency, chronic lung disease, recurrent infections, or early deaths.

On physical examination, he showed signs of respiratory distress, tachypnea and hypoxia. His height (120 cm) and weight (17 kg) were below the 3rd centile with weight far more affected than height. There were no dysmorphic features, but he had grade three clubbing of the hands and feet. The tonsils were normal and there was no lymphadenopathy. Although he had received BCG vaccine at birth, there was no evidence of BCG scar. On chest examination, there was coarse crepitation bilaterally. Other systemic examinations were unremarkable. The patient was put on broad spectrum antibiotics. CT scan of the chest showed bronchiectatic changes. Sweat chloride test was normal at two different time points (30 and 35 mmol).

**Figure 1:**
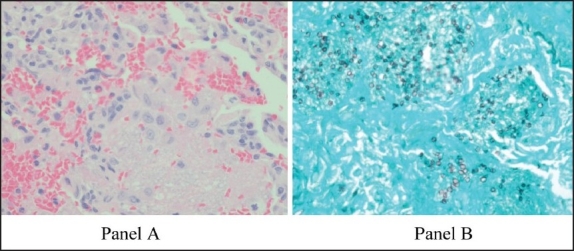
Lung biopsy from 8-year-old boy with delayed presentation of severe combined Immunodeficiency and maternal T cell engraftment. Panel A shows mild alveolar wall thickening and predominant intra-alveolar eosinophilic infiltrate; panel B shows special stain for a Giemsa stain with heavy growth of Pneumocystis jiroveci pneumonia.

Immunological findings are shown in Table 1 and 2. The high IgE level indicated normal isotype switching and thus ruled out the hyper IgM syndrome. This possibility was further excluded by the intact expression of CD40 on the surface of B lymphocytes and CD154 on CD4 T cells post PMA stimulation for 4 hours. We were not able to test the ability of B cells to mount antibody response as he was started on intravenous immunoglobulins; however, they appeared to produce reasonable pre-vaccination levels. HIV tests for both antibodies and RNA levels were negative. The adenosine deaminase B level was 1.5 IU/g Hb (normal range, 0.3-1.5 IU/g Hb). A purine nucleoside phosphorylase deficiency was unlikely with normal uric acid level (171 μmol/L; normal range, 60-240). Short tandem repeat analysis of patient peripheral blood showed 2.4% maternal T lymphocytes engraftment and 2.6% myeloid cells engraftment. The HLA typing showed full compatibility with his mother. Lung biopsy revealed eosinophilic infiltrate and a Grema stain was positive for PJP (Figure 1). Based on these data, he clearly had a T- B+ SCID phenotype with maternal T cell engraftment. Therefore, he was treated with intravenous co-trimoxazole (Septrin, GlaxoSmithKline, UK) for four weeks with a good clinical response and placed on intravenous immunoglobulin replacement therapy. HSCT was initiated. Subsequently, he was admitted again with a CMV infection confirmed by a high CMV viral load that responded well to ganciclovir. The patient was screened for mutation in *RAG1, RAG2* and Artemis genes via genotyping and direct gene sequencing, but no mutation was detected, possibly because the mutations were outside the coding regions of the screened genes, or possibly there were defects in other candidate genes, some of which might be novel, that are yet to be discovered in this patient. He was being investigated to delineate the underlying genetic defect.

**Table 1:** Immunological findings.

	Patient value	Normal age-matched value
**Immuoglobulin**		
IgG (g/L)	2.35	6-15.7
IgA (g/L)	0.49	0.45-2.3
IgM (g/L)	0.56	0.52-2.4
IgE (IU/L)	3022	<350

**Antibody response (titre)**		
Anti-tetanus toxoid IgG IU/mL	0.09	
Anti-tetanus toxoid IgG 1 mg/L	0.7	
Anti-pneumococcal IgG IU/mL	<3.30	
Anti-pneumococcal IgG 2 mg/L	<1.11	

**Lymphocyte subpopulation (×10^9^/L)**		
CD2	1600	2000-5000
CD3 (%)	943 (66%)	1700-1900
CD4 (%)	380 (28%)	800-1700
CD8 (%)	417 (30%)	700-1000
CD19 (%)	424 (28%)	400-800
CD16-56 (%)	57 (4%)	200-400

**Table 2:** In vitro proliferation assays.

Mitogen	Patient CPMa (%)	Control 1 (CPM^a^)	Control 2 (CPM^a^)
PHA	9518 (7%)	154571	1141534
Con A	8311 (10%)	86589	76009
PWM	20976 (37%)	66017	51439
Pooled allogeneic cells	3023 (4%)	44054	40543

a CPM: count per minute, PHA: phytohemagglutinin, ConA: concanavalin A, PWM: pokeweed nitrogen

The HLA typing was done on peripheral blood lymphocytes and on epithelial cells from a buccal smear. HLA class I low resolution and HLA Class II high resolution typing was performed. HLA typing was performed using sequence-specific oligonucleotides (Lifematch, Tepnel Lifecodes Molecular Diagnostics, Stamford, CT 06902, USA) and sequence specific primers (Invitrogen Corporation, Carlsbad, CA 92008, USA) described elsewhere. It revealed full matching between the patient and his mother as follows: A*02, A*26, B*49, B*50, Cw*06, Cw*07, DRB1*0701, DRB1*1301, DRB3*01/02/03, DRB4*01, DQB1*0202, DQB1*0603; and for the father: A*26, A*68, B*50, B*51, Cw*06, Cw*15

## DISCUSSION

It is well documented that maternal cells are able to cross the placenta and be detected in the healthy newborn.^6^,^7^ In an immune competent host, these maternal cells are usually rejected by the host T cell lymphocytes, but they live longer in infants with impaired cellular immunity as SCID. These cells are usually malfunctional and are not able to change the course of the disease due to their impaired or absent in vitro proliferative responses to mitogens.^7^–^9^ However, a recent report of an 8-year-old child with SCID who presented with modest recurrent infections and detectable maternal cells in the peripheral circulation indicated that such cells may provide the required immunocompetence and result in prolonged survival in patients with SCID.^10^

Although our patient presented with recurrent chest infection leading to bronchectasis, he survived until the age of 9 years, probably due to maternal T and myeloid cells engraftment enhanced by HLA compatibility with his mother. These cells are believed to play an important role in the T, B and antigen-presenting cell cooperation in view of parental consanguinity, which has been found in 56% of Saudi population.^11^ The maternal T cells in our patient were detected in a low percentage (2.4%), which probably has declined over time with apparent exhaustion of their function and inability to provide efficient immune constitution, evidenced by the significantly reduced T lymphocyte proliferation response to mitogens and predisposition to opportunistic infections as PJP and CMV.

**Figure 2:**
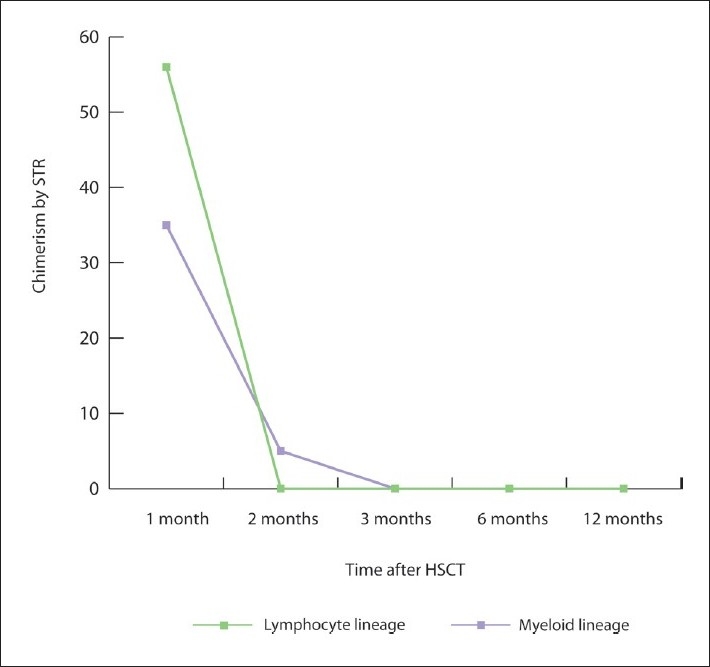
Chimeric studies after unrelated matched cord stem cell transplantation over 12 months period showing early but non-sustained engraftment. The allograft rejection is probably caused by maternal T cells. STR: short tandem repeat

Maternal T cells in SCID infants have the capacity to mediate undesired immune function despite their inability to respond efficiently to mitogens in vitro manifesting by allograft rejection and immune cytopenia, in addition to their ability to cause GVHD.^12^,^13^ In a large cohort of 121 infants with SCID undergoing HSCT, maternal T cells persisted in 48 (40%) of the patients.^8^ The majority were asymptomatic, while 19 (40%) of the 48 patients had findings consistent with GvHD, mainly involving the skin.^8^ It was shown that the low level of maternal T lymphocytes associated with low incidence of GvHD, which could explain the lack of GvHD symptoms at the time of presentation in our patient; as maternal T cells were found only at 2.4%. Our patient underwent HLA-matched unrelated cord blood stem cell transplantation. Although he engrafted initially with mixed chimerism, he eventually lost his graft (Figure 2). It has been shown that transplacentally acquired maternal T cells might cause allograft rejection and immune cytopenia, which probably explain the allograft rejection in our patient.^12^

In conclusion, transplacental maternal T cell engraftment may provide prolonged, but not persistent immunity in SCID in rare cases, and it should be suspected in late presentation of SCID. These cells usually exhaust overtime and patients should go for long-term intervention either by HSCT or gene therapy.
